# Comparison of characteristics and anti-MDA5 antibody distribution and effect between clinically amyopathic dermatomyositis and classic dermatomyositis: a retrospective case-control study

**DOI:** 10.3389/fimmu.2023.1237209

**Published:** 2023-11-27

**Authors:** Qiang Ji, Wenping Pan, Di Zhang, Yanfeng Hou, Zhankui Wang

**Affiliations:** ^1^ Department of Rheumatology and Autoimmunology, The First Affiliated Hospital of Shandong First Medical University, Shandong Provincial Qianfoshan Hospital, Shandong Key Laboratory of Rheumatic Disease and Translational Medicine, Jinan, Shandong, China; ^2^ Shandong medicine and Health Key Laboratory of Rheumatism, Jinan, Shandong, China; ^3^ First Clinical Medical College, Shandong University of Traditional Chinese Medicine, Jinan, Shandong, China; ^4^ Affiliated Hospital of Shandong University of Traditional Chinese Medicine, Jinan, Shandong, China

**Keywords:** clinically amyopathic dermatomyositis, classic dermatomyositis, anti-MDA5 antibodies, rapidly progressive interstitial lung disease, risk factors for death

## Abstract

**Background:**

Clinically amyopathic dermatomyositis (CADM) is a distinct subtype of dermatomyositis (DM) characterized by typical DM cutaneous findings but with minimal or no evidence of myositis. It possesses unique features different from classic DM (CDM). Anti-melanoma differentiation-associated gene 5 (MDA5) antibodies were found in CADM and are thought to increase the risk of rapidly progressive interstitial lung disease (RP-ILD) and are present in both CADM and CDM patients, affecting their condition and prognosis. Nevertheless, no large-sample studies have compared all aspects concerning patients with CADM and those with CDM. This study aimed to investigate differences in clinical characteristics and risk factors for mortality between CADM and CDM and to clarify the distribution and impact of anti-MDA5 antibodies in patients with these conditions.

**Methods:**

A retrospective case-control study included 330 patients and collected and analyzed their clinical data from The First Affiliated Hospital of Shandong First Medical University and Shandong Provincial Hospital of Traditional Chinese Medicine between January 2015 and July 2022; all patients were followed up to evaluate changes in their condition and prognosis. Several new cohorts were designed around anti-MDA5 antibodies to explore their distribution and impact in CADM and CDM.

**Results:**

We found CADM to be associated with higher rates of mortality, 1-year mortality, interstitial lung disease (ILD), and RP-ILD than CDM. In CADM, RP-ILD, anti-MDA5 antibodies, and high ferritin and lactate dehydrogenase (LDH) levels were identified as independent risk factors for death. In CDM, the neutrophil-to-lymphocyte ratio, anti-MDA5 antibodies, and high ferritin levels were shown to be independent risk factors for death, whereas mechanic’s hand was considered a protective factor against it. Anti-MDA5 antibody-positive patients did not exhibit any significant difference based on whether they belonged to the CADM or CDM groups. When no anti-MDA5 antibody-positive patients participated, the ferritin levels and rates of RP-ILD and ILD were still higher in CADM than in CDM; however, such differences decreased, whereas the LDH levels, rates of mortality, and 1-year mortality did not differ. Anti-MDA5 antibody-positive patients consistently showed higher LDH and ferritin levels, lower lymphocyte levels, higher probability of RP-ILD and ILD, and worse prognosis than anti-MDA5 antibody-negative patients, irrespective of whether the patients had DM, CADM, or CDM.

**Conclusion:**

Patients with CADM exhibit relatively worse symptoms, serological findings, and prognosis than those with CDM. Furthermore, patients with CADM and those with CDM have commonalities and differences in risk factors for death. Moreover, CADM may necessitate earlier and more aggressive treatment strategies than CDM. Anti-MDA5 antibodies occur at a high level in patients with CADM, not only affecting the symptoms and prognosis of DM but also having a non-negligible impact on the differences between CADM and CDM. Hence, screening for anti-MDA5 antibodies in patients with CADM and CDM is extremely essential.

## 1 Introduction

Idiopathic inflammatory myopathy is a heterogeneous group of diseases characterized by inflammation affecting the skeletal muscles and extramuscular organs, particularly the skin and lungs (1, 2). The most common clinical subtypes of idiopathic inflammatory myopathy in adults are polymyositis and dermatomyositis (DM) (3, 4). First proposed by Euwer and Sontheimer as a subcategory of idiopathic inflammatory myopathy (5), amyopathic dermatomyositis is characterized by the hallmark cutaneous manifestations of DM and the absence of any clinical or laboratory evidence of muscle disease for ≥6 months (6). Clinically amyopathic dermatomyositis (CADM) can be divided into amyopathic DM and hypomyopathic DM (7). Hypomyopathic DM is defined as the presence of cutaneous lesions consistent with DM and in the absence of overt muscle weakness despite laboratory, electrophysiological, and radiologic evidence of muscle disease. Although, the absence of clinically evident muscle diseases in CADM may differentiate it from classic dermatomyositis (CDM), distinguishing the cutaneous manifestations of ADM from those of CDM has not been possible to date.

Dermatomyositis damages not only the skin and muscles but also other organs. Interstitial lung disease (ILD), malignancy, and myocardial involvement are its relatively common extramuscular findings. Of them, ILD is considered a common severe complication, with a reported prevalence of 5–65% (8, 9). The disease course and severity of ILD are highly heterogeneous (10), wherein some patients with mild ILD respond markedly to treatment without exacerbation, whereas others are at a risk of developing rapidly progressive ILD (RP-ILD), which is often insensitive to treatment and has a poor prognosis (11, 12).

Myositis-specific antibodies are present in 50–70% of all DM patients and are associated with distinct clinical features (13). Moreover, Incorporating them into myositis diagnostic algorithms could better define the clinical phenotype, prognosis, and treatment response of patient subgroups. Anti-melanoma differentiation-associated gene 5 (MDA5) antibodies were originally identified in patients with CADM who had predominantly prolonged skin lesions without accompanying muscle weakness and were at risk of progressing to acute RP-ILD (14). Previous reports have indicated that, depending on the cohort (14–22), 23–100% of anti-MDA5 antibody-positive patients develop CADM and have shown that anti-MDA5 antibody-associated ILD rapidly progresses and has a poor prognosis (23).

Several recent studies on DM have focused on the characteristics and poor prognosis of patients with anti-MDA5 antibodies in the CADM cohort. Nonetheless, since CADM was first defined, no large-sample studies have compared all aspects of patients with CADM and those with CDM. Bowerman et al. (24) conducted a retrospective cohort study involving 201 patients with adult-onset DM. However, their main research focus was on the prevalence of tumors in patients with ADM and CDM and on the factors affecting these tumors. The clinical manifestations, serological manifestations, specific autoantibody types, prognosis, and distribution of patients with anti-MDA5 antibody-associated DM differ between CADM and CDM. Accordingly, the present retrospective study collected and analyzed the clinical data of inpatients with CADM and those with CDM from The First Affiliated Hospital of Shandong First Medical University and Shandong Provincial Hospital of Traditional Chinese Medicine between January 2015 and July 2022. All patients were followed up to evaluate changes in their condition and prognosis. Several new cohorts were designed around anti-MDA5 antibodies to explore the distribution and impact of anti-MDA5 antibodies in patients with CADM and CDM and to clarify differences in clinical, treatment-related, and prognostic features between patients with CADM and those with CDM. As far as we know, this is the only cohort study that includes more than 300 patients for comparison between CADM and CDM.

## 2 Materials and methods

### 2.1 Study participants

The study participants were patients diagnosed with DM at The First Affiliated Hospital of Shandong First Medical University and Shandong Provincial Hospital of Traditional Chinese Medicine (The hospitals are located at No. 16766 Jingshi Road and No. 42 Wenhua West Road, Lixia District, Jinan City, Shandong Province, China) between January 2015 and July 2022. Patients whose main condition treated or investigated during hospitalization was not DM were excluded from the analysis. This study was conducted in accordance with the principles embodied in the Declaration of Helsinki and was approved by the Human Research Ethics Boards of The First Affiliated Hospital of Shandong First Medical University (approval no.: YXLL-KY-2020-025) and Shandong Provincial Hospital of Traditional Chinese Medicine (approval no.: 2021-027-KY). Informed consent was obtained from all donors prior to their inclusion in the study, and all patient data were anonymized.

### 2.2 Data collection

Clinical data collected included demographics, clinical characteristics, and laboratory results. In our cohorts, lymphocyte subset analyses were conducted on 270 patients on their first visit. Although some patients had not previously received glucocorticoid therapy, numerous ones received this therapy at other hospitals for a short period, which might have fluctuated the predictive accuracy of the lymphocyte subsets. The levels of tumor markers, including carcinoembryonic antigen (CEA), alpha-fetoprotein, carbohydrate antigen (CA) 19-9, CA125, CA153, CA724, CA50, and CA242, neuron-specific enolase (NSE), CYFRA21, and squamous cell carcinoma antigen of the 270 patients were measured. Out of 330 patients, 74 underwent pulmonary function tests. We recorded the results of the pulmonary function tests, including the forced vital capacity, forced expiratory volume in 1 s, and diffusing capacity of the lungs for carbon monoxide. Myositis autoantibody and all routine tests were performed on all the patients in our cohorts during their first visit. Serum antinuclear antibody profiles and myositis-specific antibodies were detected by a third-party testing company (EUROIMMUN Medical Laboratory Diagnostics Stock Company, China) according to the manufacturer’s instructions (Euroimmun, Germany). The tested antigens were Mi-2α, Mi-2β, TIF1γ, MDA5, NXP2, SAE1, Ku, PM-Scl100, PMScl75, Jo-1, SRP, PL-7, PL-12, EJ, OJ, Ro-52, cN-1A, Ha, Ks and ZO. The cut-offs values for the results were 0–5 (negative), 6-10 (borderline), 11–25 (+) and 26-50 (++), strong positive (+++). Because both the anti-Mi-2α and Mi-2β antigens target two closely related isoforms of the same protein, they were considered together as anti-Mi-2. In this study antibody positivity was defined by a blot intensity of ≥25. We excluded the weakly positive specific autoantibodies in patients who had multiple positive autoantibodies. Moreover, the values for these antibodies might change during follow-up, wherein a patients with double positive antibody values might show single positive values on later testing. Additionally, the neutrophil-to-lymphocyte ratio (NLR) and platelet-to-lymphocyte ratio (PLR) were determined. In our cohorts, the follow-up start point was the patient’s first visit, and the follow-up end point was April 30, 2023 or the time patient’s death.

### 2.3 Diagnostic criteria

We diagnosed DM according to the Bohan and Peter criteria and based on the 239^th^ European Neuromuscular Centre international workshop guidelines (25, 26). We categorized ILD into RP-ILD or chronic ILD according to its clinical manifestations. Additionally, RP-ILD was defined as the presence of two or more of the following within 3 months: (i) exacerbation of dyspnea; (ii) an increase in parenchymal abnormalities on high-resolution computed tomography; and (iii) either a decrease of >10% in vital capacity or a decrease of >1.33 kPa in arterial oxygen tension (PaO_2_). Chronic ILD was defined as a slowly of the progressive ILD exhibiting gradual deterioration over 3 months (27).

### 2.4 Cohort design

The cohorts were established based on the anti-MDA5 antibodies to clarify differences in clinical, treatment-related, and prognostic features between patients with CADM and those with CDM along with investigating the distribution and impact of anti-MDA5 antibody-associated DM in patients with CADM and those with CDM. The DM cohort was established to elucidate the differences between patients with CADM (CADM group) and those with CDM (CDM group). The DM-MDA5 cohort was established to evaluate the differences between anti-MDA5 antibody-positive patients (MDA5+ group) and anti-MDA5 antibody-negative patients (MDA5− group). The CADM cohort was established to explore the differences between patients with anti-MDA5 antibody-positive CADM (CADM-MDA5+ group) and those with anti-MDA5 antibody-negative CADM (CADM-MDA5− group). The CDM cohort was established to elucidate the differences between anti-MDA5 antibody-positive patients with CDM (CDM-MDA5+ group) and anti-MDA5 antibody-negative patients with CDM (CDM-MDA5− group). The DM-MDA5+ cohort was established to assess the differences between anti-MDA5 antibody-positive patients with CADM (CADM-MDA5+ group) and anti-MDA5 antibody-positive patients with CDM (CDM-MDA5+ group). The DM-MDA5- cohort was established to examine the differences between anti-MDA5 antibody-negative patients with CADM (CADM-MDA5− group) and anti-MDA5 antibody-negative patients with CDM (CDM-MDA5− group).

### 2.5 Statistical analysis

Qualitative data are presented as numbers and percentages, whereas quantitative data are expressed as means and standard deviations or as medians and interquartile ranges, depending on the skewness of data. Regarding group comparisons of various data types, we used the χ^2^ test to examine categorical data, while the two-sample *t*-test or Mann–Whitney *U* test was used to analyze continuous data. We determined the optimal cut-off value for death by receiver operating characteristic curve analysis and transformed each continuous parameter into a categorical variable. We built multivariable Cox proportional hazards models to identify the independent prognostic risk factors and to calculate their hazard ratios (HRs), 95% confidence intervals (CIs), and β regression coefficients. The independent risk factors for death in CADM and CDM were evaluated using backward stepwise selection with Cox regression. As for time-event analysis, the cumulative survival rates during the follow-up were calculated using the Kaplan–Meier method, and the different groups were compared using the log-rank test. Statistical significance was set at a two-tailed *P*-value of <0.05. All statistical analyses were performed using SPSS software version 26 (IBM Corp., Armonk, NY, USA).

## 3 Results

### 3.1 Clinical characteristics of all patients

The demographic data, clinical manifestations, and laboratory test results of the 330 enrolled patients with DM at the time of diagnosis are summarized in **Table 1**. The mean patient age at the time of diagnosis was 54.40 ± 12.02 years (range: 15–81 years), and the median duration of symptoms prior to diagnosis was 3 months. Of the enrolled patients, 230 (69.7%) were women. Furthermore, 135 (40.9%) and 195 (59.1%) out of 330 patients had CADM and CDM, respectively. Overall, 243 (73.6%) patients developed ILD, whereas 29 (8.8%) exhibited an RP pattern. The most common skin lesions were Gottron’s sign and heliotrope rash, and a malignancy was detected in 25 (7.6%) patients. Myositis autoantibody tests were performed on 313 (94.8%) patients at their first admission, and 265 (84.7%) patients tested positive (including 69 [26.0%] with anti-MDA5 antibodies). All patients received glucocorticoid therapy; however, 31 (9.4%) received pulse-dose therapy. Additionally, 231 (70.0%) were treated with immunosuppressants such as cyclosporine A, tacrolimus, cyclophosphamide, azathioprine, methotrexate, and mycophenolate mofetil. Among the patients, 57 (17.3%) were found to have died owing to exacerbations of ILD or infection at follow-up, whereas 44 (13.3%) died within 1 year. The mortality and 1-year mortality rates were 44.9% and 40.6%, respectively, in anti-MDA5 antibody-positive patients.

**Table 1:** Clinical differences between clinically amyopathic dermatomyositis and classic dermatomyositis.

	DM cohort (*N*=330)	CADM (*N*=135)	CDM (*N*=195)	*P*-value
Age at diagnosis, mean ± SD, years	54.40 ± 12.02	55.05 ± 11.82	53.94 ± 12.16	0.411
Disease duration at diagnosis, median (IQR), months	3.00 (1.00, 7.00)	3.00 (2.00, 7.00)	3.00 (1.00, 7.00)	0.543
Male/female	100/230 (1:2.30)	45/90 (1:2.00)	55/140 (1:2.55)	0.319
Death, *n* (%)	57 (17.3)	35 (25.9)	22 (11.3)	0.001
Died within 1 year, *n* (%)	44 (13.3)	27 (20.0)	17 (8.7)	0.003
RP-ILD, *n* (%)	29 (8.8)	23 (17.0)	6 (3.1)	<0.001
ILD, *n* (%)	243 (73.6)	121 (89.6)	122 (62.6)	<0.001
Fever, *n* (%)	101 (30.6)	41 (30.4)	60 (30.8)	0.938
Cough or dyspnea, *n* (%)	227 (68.8)	115 (85.2)	112 (57.4)	<0.001
Mechanic’s hand, *n* (%)	104 (31.5)	37 (27.4)	67 (34.4)	0.181
Heliotrope rash, *n* (%)	143 (43.3)	55 (40.7)	88 (45.1)	0.429
Gottron’s sign, *n* (%)	233 (70.6)	105 (77.8)	128 (65.6)	0.017
V sign, *n* (%)	117 (35.5)	39 (28.9)	78 (40.0)	0.038
Shawl sign, *n* (%)	64 (19.4)	18 (13.3)	46 (23.6)	0.021
Holster sign, *n* (%)	38 (11.5)	13 (9.6)	25 (12.8)	0.372
Arthritis/arthralgia, *n* (%)	137 (41.5)	49 (36.3)	88 (45.1)	0.109
Raynaud’s phenomenon, *n* (%)	34 (10.3)	14 (10.4)	20 (10.3)	0.973
Dysphagia, *n* (%)	33 (10.0)	5 (3.7)	28 (14.4)	0.002
Gastroesophageal reflux, *n* (%)	15 (4.5)	6 (4.4)	9 (4.6)	0.942
Dry eyes, *n* (%)	25 (7.6)	11 (8.1)	14 (7.2)	0.744
Dry mouth, *n* (%)	56 (17.0)	27 (20.0)	29 (14.9)	0.222
Cardiac involvement, *n* (%)	72 (21.8)	40 (29.6)	32 (16.4)	0.004
Pleural effusion, *n* (%)	41 (12.4)	24 (17.8)	17 (8.7)	0.014
Pericardial effusion, *n* (%)	32 (9.7)	16 (11.9)	16 (8.2)	0.271
Fatigue, *n* (%)	98 (29.7)	39 (28.9)	59 (30.3)	0.789
Hoarseness, *n* (%)	28 (8.5)	20 (14.8)	8 (4.1)	0.001
Tumor, *n* (%)	25 (7.6)	9 (6.7)	16 (8.2)	0.604
Numbness in the extremities	20 (6.1)	14 (10.4)	6 (3.1)	0.006
Anti-MDA5 antibodies, *n* (%)	69 (21.1)	49 (37.4)	20 (11.0)	<0.001
LYM, median (IQR), ×10^9^/L	1.12 (0.72, 1.57)	0.81 (0.61, 1.10)	1.38 (1.00, 1.91)	<0.001
WBC count, median (IQR), ×10^9^/L	7.56 (5.57, 10.25)	7.64 (5.74, 10.55)	6.98 (4.94, 10.39)	0.373
NEUT, median (IQR), ×10^9^/L	5.45 (3.8, 7.95)	6.30 (4.32, 8.39)	5.19 (3.30, 8.06)	0.012
NLR, median (IQR)	4.48 (2.84, 8.33)	7.14 (4.40, 12.05)	3.46 (2.42, 5.65)	<0.001
PLT, median (IQR), ×10^9^/L	226.50 (177.00, 293.25)	227.00 (165.25, 297.75)	236.00 (186.25, 289.75)	0.711
PLR, median (IQR)	199.52 (132.99, 304.62)	265.79 (205.63, 379.25)	172.06 (115.59,276.07)	<0.001
LDH, median (IQR), U/L	284.50 (207.75, 393.00)	307.00 (221.25, 429.50)	275.80 (208.75, 364.00)	0.004
α-HBDH, median (IQR), U/L	206.00 (142.75, 301.50)	199.50 (154.25, 283.50)	243.00 (170.25, 350.00)	0.677
Serum ferritin, median (IQR), ng/mL	258.00 (134.55, 620.75)	361.30 (159.30, 885.82)	239.03 (123.10, 550.80)	<0.001
CRP, median (IQR), mg/L	6.20 (3.19, 20.60)	8.45 (3.30, 27.00)	4.10 (3.12, 14.08)	0.002
ESR, median (IQR), mm/h	24.00 (14.00, 44.25)	26.5 (16.00, 43.25)	23 (14.00, 41.50)	0.111
Procalcitonin, median (IQR), mg/L	0.05 (0.04, 0.13)	0.05 (0.04, 0.12)	0.05 (0.04, 0.08)	0.268
D-dimer, median (IQR), mg/L	0.60 (0.29, 1.29)	0.56 (0.30, 1.26)	0.69 (0.28, 1.31)	0.582
LYM ≤785×10^6^/L, *n* (%)	102 (30.9)	67 (49.6)	35 (17.9)	<0.001
NLR ≥6.83, *n* (%)	110 (33.3)	72 (53.3)	38 (19.5)	<0.001
LDH ≥354.50 U/L, *n* (%)	112 (33.9)	58 (4.3)	54 (27.7)	0.004
Serum ferritin ≥649.95 ng/mL, *n* (%)	78 (23.6)	48 (35.6)	30 (15.4)	<0.001

ILD, interstitial lung disease; RP-ILD, rapidly progressive interstitial lung disease; LYM, lymphocyte; WBC, white blood cell; NEUT, neutrophil; NLR, NEUT-to-LYM ratio; PLT, platelet; PLR, PLT-to-LYM ratio; LDH, lactate dehydrogenase; α-HBDH, α-hydroxybutyrate dehydrogenase; CRP, C-reactive protein; ESR, erythrocyte sedimentation rate; IQR, interquartile range; CADM, clinically amyopathic dermatomyositis; CDM, classic dermatomyositis.

### 3.2 Comparison of clinical characteristics between patients with CADM and those with CDM

We compared the clinical characteristics of the two groups of patients, and the comparison results are shown in **Table 1**. Patients with CADM had higher rates of death (25.9% vs. 11.3%, *P*=0.001), 1-year mortality (20.0% vs. 8.7%, *P*=0.003), ILD (89.6% vs. 62.6%, *P*<0.001), RP-ILD (17.0% vs. 3.1%, *P*<0.001), cough or dyspnea (85.2% vs. 57.4%, *P*<0.001), Gottron’s sign (77.8% vs. 65.6%, *P*=0.026), cardiac involvement (29.6% vs. 16.4%, *P*=0.004), pleural effusion (17.8% vs. 8.7%, *P*=0.014), hoarseness (14.8% vs. 4.1%, *P*=0.001), and numbness in the extremities (10.4% vs. 3.1%, *P*=0.006) than patients with CDM. Furthermore, patients with CADM exhibited lower rates of the V sign (28.9% vs. 40.0%, *P*=0.038), shawl sign (13.3% vs. 23.6%, *P*=0.021), and dysphagia (3.7% vs. 14.4%, *P*=0.002) than patients with CDM. The two groups showed similar rates for clinical symptoms such as mechanic’s hand, heliotrope rash, V sign, Holster sign, arthritis/arthralgia, Raynaud’s phenomenon, gastroesophageal reflux, dry eyes, dry mouth, pleural effusion, pericardial effusion, fatigue, and tumors.

With respect to serological indices, the lymphocyte levels in the CADM group were significantly lower than those in the CDM group (0.81 vs. 1.38 ×10^9^/L, *P*<0.001). The NLR (7.14 vs. 3.46, *P*<0.001), PLR (265.79 vs. 172.06, *P*<0.001), and neutrophil (6.30 vs. 5.19×10^9^/L, *P*=0.012), lactate dehydrogenase (LDH) (307.00 vs. 275.80 U/L, *P*=0.004), ferritin (361.30 vs. 239.03 ng/mL, *P*<0.001), and C-reactive protein (CRP) levels (8.45 vs. 4.10 mg/L, *P*=0.002) in the CADM group were significantly higher than those in the CDM group.

**Figure 1:**
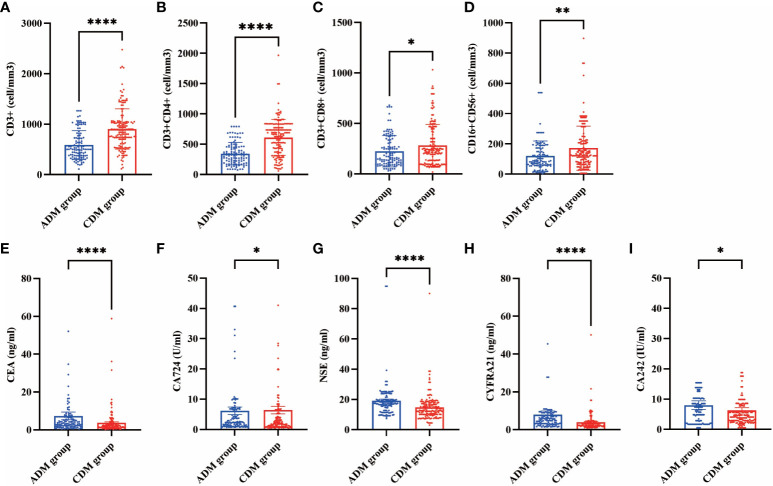
Differences in lymphocyte subsets (**A**, CD3+ **B**, CD3+CD4+ **C**, CD3+CD8+ **D**, CD16+CD56+) and tumor markers (**E**, CEA **F**, CA724 **G**, NSE **H**, CYFRA21 **I**, CA242) levels between the CADM and CDM groups. (*, P≤ 0.05 **, P≤ 0.01 ****, P≤ 0.0001).

In our cohorts, lymphocyte subset analyses were conducted on a total of 270 patients at their first visit (**Supplementary Table 1**). The CD3+ (502.00 vs. 882.65 cells/mm^3^, *P*<0.001), CD3+CD4+ (324.48 vs. 670.45 cells/mm^3^, *P*<0.001), CD3+CD8+ (162.69 vs. 229.60 cells/mm^3^, *P*=0.048), and CD16+CD56+ (89.00 vs. 125.30 cells/mm^3^, *P*=0.002) cell counts in the CADM group were significantly lower than those in the CDM group. Tumor marker levels were also measured in these 270 patients (**Supplementary Table 2**). the serum CEA (3.30 vs. 2.06 ng/mL, *P*<0.001), CA724 (2.43 vs. 1.82 U/mL, *P*=0.015), NSE (17.11 vs. 13.02 ng/mL, *P*<0.001), CYFRA21 (4.82 vs. 3.18 ng/mL, *P*<0.001), and CA242 (6.50 vs. 4.10 U/mL, *P*=0.024) levels in the CADM group were significantly higher than those in the CDM group. The differences in lymphocyte subsets and tumor marker levels between the CADM and CDM groups are shown in **Figure 1**.

The distribution of myositis-specific autoantibodies in patients with CADM and those with CDM is shown in **Table 2**. Patients with CDM had higher levels of negative myositis-specific autoantibodies (20.9% vs. 7.6%, *P*=0.001) and anti-NXP2 antibodies (7.7% vs. 1.5%, *P*=0.029) than those with CADM. However, the CADM group exhibited higher levels of anti-MDA5 antibodies (37.4% vs. 11.0%, *P*<0.001) than the CDM group.

**Table 2:** Comparison of myositis-specific autoantibodies between clinically amyopathic dermatomyositis (CADM) and classic dermatomyositis (CDM).

Myositis-specific autoantibodies	CADM group (*N*=131)	CDM group (*N*=182)	*P*-value
Negative	10 (7.6%)	38 (20.9%)	0.001
Anti-JO-1	21 (16.0%)	45 (24.7%)	0.063
Anti-PL-7	14 (10.7%)	10 (5.5%)	0.089
Anti-PL-12	9 (6.9%)	3 (1.6%)	0.038
Anti-HA	1 (0.8%)	0 (0%)	0.419
Anti-EJ	10 (7.6%)	14 (7.7%)	0.985
Anti-OJ	1 (0.8%)	0 (0%)	0.419
Anti-Mi-2	3 (2.3%)	10 (5.5%)	0.265
Anti-MDA5	49 (37.4%)	20 (11.0%)	<0.001
Anti-NXP2	2 (1.5%)	14 (7.7%)	0.029
Anti-TIF-1γ	8 (6.1%)	22 (12.1%)	0.076
Anti-SAE1	3 (2.3%)	2 (1.1%)	0.710
Anti-SPR	0 (0%)	4 (2.2%)	0.143

Out of 330 patients, 74 underwent pulmonary function tests. The forced vital capacity, forced expiratory volume in 1 s, and diffusing capacity of the lungs for carbon monoxide were lower in the CADM group than those in the CDM group, albeit without statistically significant differences. Nevertheless, because some patients with dyspnea were unable to undergo pulmonary function tests, we believe that the recorded pulmonary function test results were not representative of the true level in patients with DM. A comparison of pulmonary function test results for each cohort is presented in **Supplementary Table 3**.

### 3.3 Risk factors independently associated with prognosis in patients with CADM and those with CDM

**Figure 2:**
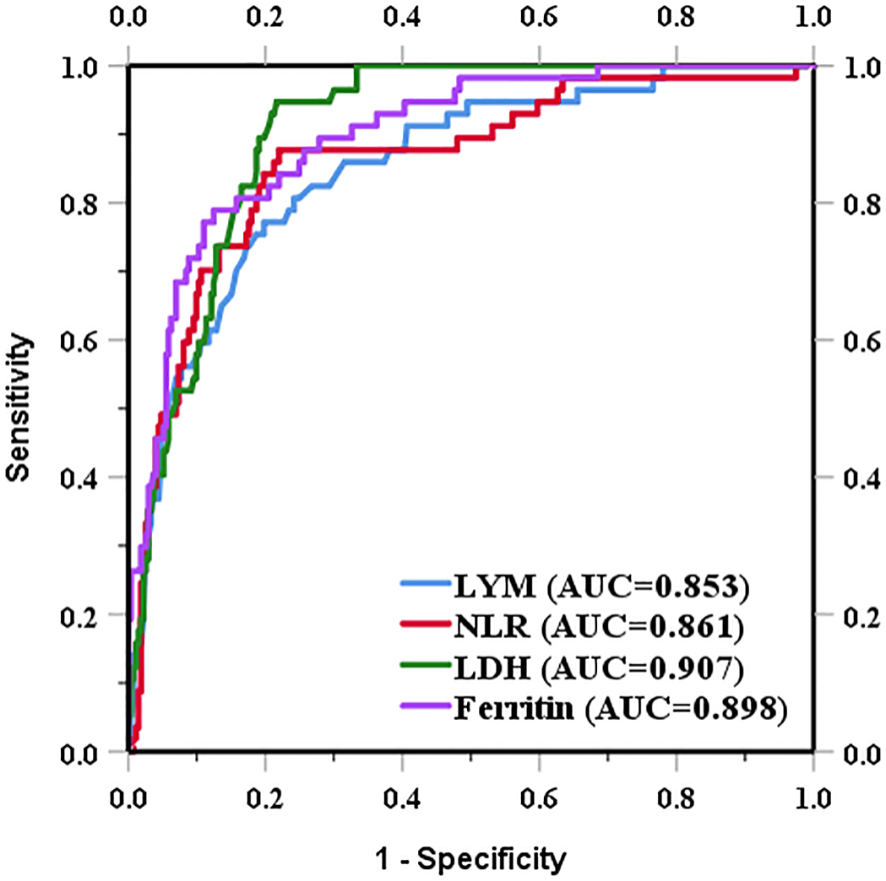
Prognostic value of the NLR and the levels of LDH, ferritin, and lymphocytes in DM.

We determined the optimal cut-off value for death by receiver operating characteristic curve analysis and transformed each continuous parameter into a categorical variable. The optimal cut-off points for NLR, LDH, ferritin, and lymphocytes were 6.83 (area under the curve [AUC], 0.861; sensitivity, 88.7%; specificity, 78.0%), 354.50 U/L (AUC, 0.907; sensitivity, 94.7%; specificity, 78.4%), 649.95 ng/mL (AUC, 0.898; sensitivity, 78.9%; specificity, 87.5%), and 785.00×10^6^/L (AUC, 0.853; sensitivity, 77.2%; specificity, 80.2%), respectively (**Figure 2**).

**Figure 3:**
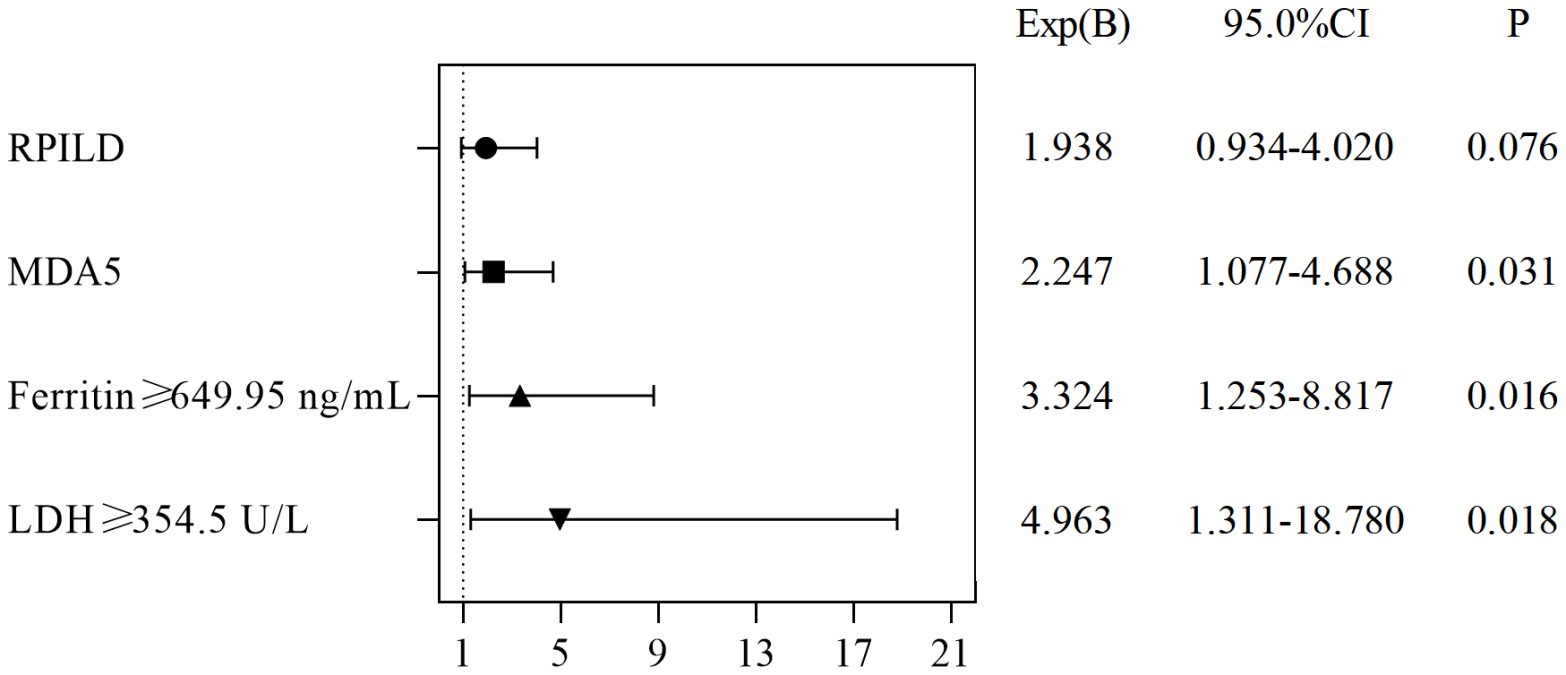
Risk factors for mortality in patients with CADM.

**Figure 4:**
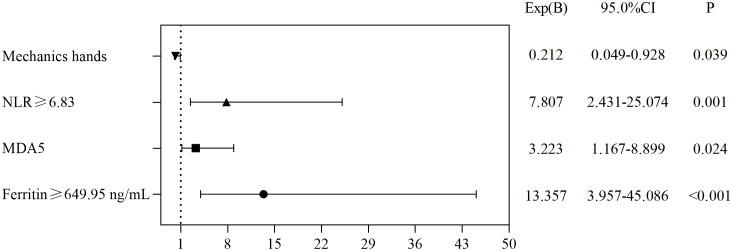
Risk factors for mortality in patients with CDM.

The independent risk factors for death in CADM and CDM were evaluated using backward stepwise selection with Cox regression. Multivariate Cox analysis revealed that RP-ILD (HR, 1.938; 95% CI, 0.934–4.020; *P*=0.076), anti-MDA5 antibodies (HR, 2.247; 95% CI, 1.077–4.688; *P*=0.031), ferritin levels ≥649.95 ng/mL (HR, 3.324; 95% CI, 1.253–8.817; *P*=0.016), and LDH levels ≥354.5 U/L (HR, 4.963; 95% CI, 1.311–18.78; *P*=0.018) were independent risk factors for death in CADM (**Figure 3**), whereas NLR ≥6.83 (HR, 7.807; 95% CI, 2.431–25.074; *P*=0.001), anti-MDA5 antibodies (HR, 3.223; 95% CI, 1.167–8.899; *P*=0.024), and ferritin levels ≥649.95 ng/mL (HR, 13.357; 95% CI, 3.957–45.086; *P*<0.001) were independent risk factors for death in CDM. Notably, mechanic’s hand was considered a protective factor against death in CDM (HR, 0.212; 95% CI, 0.049–0.928; *P*=0.039) (**Figure 4**).

### 3.4 Clinical treatment and treatment response

The responses to clinical therapy and treatment in the 330 patients are detailed in **Table 3**. During treatment, intravenous immunoglobulin (IVIG) was administered to 27.9% of patients, whereas biological agents were used in 6.1% of patients. Patients with CADM had significantly lower rates of only using oral corticosteroid (3.7% vs. 14.9%, *P*=0.001) and higher rates of cyclosporine A use (16.3% vs. 6.2%, *P*=0.003), corticosteroid (IV and/or oral) + cyclosporine A or tacrolimus + cyclophosphamide use (16.3% vs. 8.2%, *P*=0.024), corticosteroid pulse-dose therapy (15.6% vs. 5.2%, *P*=0.001), IVIG use (40.7% vs. 19.0%, *P*<0.001), and biological agent use (9.6% vs. 3.6%, *P*=0.024) than patients with CDM. With respect to ILD’s response to treatment, patients with CADM showed a higher rate of worsening (44.3% vs. 24.4%, *P*=0.001) and a lower rate of improvement (13.9% vs. 35.6%, *P*<0.001) than patients with CDM.

**Table 3:** Treatment and clinical course in the entire cohort.

Characteristic	CADM	CDM	*P*-value
Initial treatment			
CS (oral only)	5 (3.7%)	29 (14.9%)	0.001
CS (IV + oral only)	28 (20.7%)	37 (19.0%)	0.692
CS (IV and/or oral) + CsA	22 (16.3%)	12 (6.2%)	0.003
CS (IV and/or oral) + Tac	29 (21.5%)	26 (13.3%)	0.051
CS (IV and/or oral) + CY (oral and/or IV)	6 (4.4%)	16 (8.2%)	0.178
CS (IV and/or oral) + AZA	2 (1.5%)	5 (2.6%)	0.778
CS (IV and/or oral) + MTX	1 (0.7%)	9 (4.6%)	0.091
CS (IV and/or oral) + MMF	22 (16.3%)	44 (22.6%)	0.162
CS (IV and/or oral) + CsA or Tac + CY (oral and/or IV)	22 (16.3%)	16 (8.2%)	0.024
CS pulse-dose	21 (15.6%)	10 (5.2%)	0.001
IVIG	55 (40.7%)	37 (19.0%)	<0.001
JAK inhibitors	7 (5.2%)	7 (3.6%)	0.480
Biological agents	13 (9.6%)	7 (3.6%)	0.024
ILD response to treatment			
Worsening	54 (44.3%)	33 (24.4%)	0.001
Stability	45 (36.9%)	41 (30.4%)	0.269
Improved	17 13.9%)	48 (35.6%)	<0.001
Unknown	6 (4.9%)	13 (9.6%)	0.149

CS, corticosteroid; CsA, cyclosporine A; Tac, tacrolimus; CY, cyclophosphamide; IV, intravenous; IVIG, intravenous immunoglobulin; AZA, azathioprine; MTX, methotrexate; MMF, mycophenolate mofetil; CS pulse-dose, 0.5–1.0 g per day for 1–5 days; CADM, clinically amyopathic dermatomyositis; CDM, classic dermatomyositis.

### 3.5 Impact of anti-MDA5 antibodies on CADM and CDM

The positivity rates for anti-MDA5 antibodies in patients with CADM and those with CDM were 37.4% and 11.0%, respectively. Clinical characteristics and serological indices were compared between patients with CADM and those with CDM who tested positive for anti-MDA5 antibodies to explore the differences in anti-MDA5 antibodies between CADM and CDM. We found that only the incidence of the V sign was significantly lower in the CADM-MDA5+ group than in the CDM-MDA5+ group (*P*=0.032). However, the differences in the remaining characteristics were not statistically significant.

Differences between CADM and CDM in patients who tested negative for anti-MDA5 antibodies were also analyzed. After excluding MDA5 patients, the rates of RP-ILD (*P*=0.005), ILD (*P*<0.001), cough or dyspnea (*P*=0.001), cardiac involvement (*P*=0.029), the numbness in the extremities (*P*=0.028), the NLR (*P*=0.001), PLR (*P*=0.001), and the levels of ferritin (*P*=0.003), CRP (*P*=0.025), NSE (*P*=0.002), and CYFRA21 (*P*<0.001) in the CADM-MDA5− group were still higher than those in the CDM-MDA5− group. Furthermore, the rates of the shawl sign (*P*=0.025) and dysphagia (*P*=0.028) and the levels of lymphocytes (*P*=0.001) in the CADM-MDA5− group were still lower than those in the CDM-MDA5− group. Notably, the differences in RP-ILD, cough or dyspnea, cardiac involvement, NLR, ferritin levels, and lymphocyte levels between the two groups decreased. Furthermore, the differences in the rates of mortality, 1-year mortality, Gottron’s sign, V sign, pleural effusion, hoarseness and in the levels of LDH, CEA, CA724, and CA242 were no longer statistically significant. Clearly, when anti-MDA5 antibodies were involved, some symptoms and serological indicators differed across CADM and CDM, becoming substantial, or even changing from “nothing” to “something.”

Differences in the clinical characteristics and serological indices were also examined between anti-MDA5 antibody-positive and anti-MDA5 antibody-negative patients with DM, those with CADM, and those with CDM to evaluate the impact of anti-MDA5 antibodies on patients with DM. Whether in DM, CADM, or CDM, anti-MDA5 antibody-positive patients had consistently significantly higher rates of mortality, 1-year mortality, RP-ILD, ILD, fever, cough or dyspnea, heliotrope rashes, higher PLR, and higher levels of LDH, ferritin, and CEA than anti-MDA5 antibody-negative patients. Conversely, the disease duration at diagnosis and lymphocyte levels were consistently significantly lower than those in anti-MDA5-negative patients. However, anti-MDA5 antibody-positive patients had consistently significantly lower lymphocyte levels and disease duration at diagnosis. Variables with *P*<0.05 in the above differential analysis are shown in **Table 4**.

**Table 4:** *P*-value after the analysis of each cohort.

	*P*-value (CADM vs. CDM)	*P*-value (CADM-MDA5+ vs. CDM-MDA5+)	*P*-value (CADM-MDA5- vs. CDM-MDA5-)	*P*-value (MDA5+ vs. MDA5-)	*P*-value (CADM-MDA5+ vs. CADM-MDA5-)	*P*-value (CDM-MDA5+ vs. CDM-MDA5-)
Disease duration at diagnosis	0.543	0.157	0.198	<0.001^-^	<0.001^-^	0.001^-^
Death	0.001^+^	0.599	0.152	<0.001^+^	<0.001^+^	<0.001^+^
Died within 1 year	0.003^+^	0.950	0.374	<0.001^+^	<0.001^+^	<0.001^+^
RP-ILD	<0.001^+^	0.550	0.005^+^	<0.001^+^	0.002^+^	<0.001^+^
ILD	<0.001^+^	0.412	<0.001^+^	<0.001^+^	0.013^+^	0.028^+^
Fever	0.938	0.588	0.445	0.006^+^	0.027^+^	0.056
Cough or dyspnea	<0.001^+^	0.282	0.001^+^	<0.001^+^	0.006^+^	0.029^+^
Mechanic’s hand	0.181	0.200	0.656	0.022^-^	0.038^-^	0.608
Heliotrope rash	0.429	0.364	0.237	0.011^+^	0.033^+^	0.051
Gottron’s sign	0.017^+^	0.534	0.102	0.115	0.516	0.453
V sign	0.038^-^	0.032^-^	0.058	0.054	0.132	0.014^+^
Shawl sign	0.021^-^	0.988	0.025^-^	0.783	0.378	0.998
Holster sign	0.372	0.534	0.084	0.014^+^	0.028^+^	0.098
Raynaud’s phenomenon	0.973	1.000	0.520	0.034^-^	0.110	0.137
Dysphagia	0.002^-^	0.128	0.028^-^	0.218	0.727	1.000
Cardiac involvement	0.004^+^	0.491	0.029^+^	0.001^+^	0.113	0.068
Pleural effusion	0.014^-^	0.135	0.556	0.001^+^	0.005^+^	1.000
Pericardial effusion	0.271	0.534	0.493	0.001^+^	0.096	0.004^+^
Fatigue	0.789	0.119	0.875	0.036^+^	0.341	0.013^+^
Hoarseness	0.001^+^	0.233	0.732	<0.001^+^	<0.001+	0.061
Tumor	0.604	0.290	0.671	0.044^-^	0.026^-^	0.829
Numbness in the extremities	0.006^+^	0.822	0.028^+^	0.301	0.934	1.000
LYM	<0.001^-^	0.588	0.001^-^	0.001^-^	<0.001^-^	<0.001^-^
NLR	<0.001^+^	0.116	0.001^+^	0.001^+^	0.109	0.010^+^
PLR	<0.001^+^	0.390	0.001^+^	0.001^+^	0.022^+^	0.001^+^
LDH	0.004^+^	0.552	0.184	0.001^+^	0.002^+^	0.023^+^
Serum ferritin	<0.001^+^	0.341	0.003^+^	0.001^+^	<0.001^+^	0.002^+^
CRP	0.002^+^	0.084	0.025^+^	0.205	0.498	0.726
ESR	0.111	0.900	0.326	0.026+	0.210	0.180
D-dimer	0.582	0.969	0.045^-^	0.013^+^	0.001^+^	0.524
LYM ≤785×10^6^/L	<0.001^+^	0.523	<0.001^+^	<0.001^+^	0.016^+^	<0.001^+^
NLR ≥6.83	<0.001^+^	0.309	<0.001^+^	<0.001^+^	0.081	0.001^+^
LDH ≥354.50 U/L	0.004^+^	0.309	0.398	<0.001^+^	<0.001^+^	0.029^+^
Serum ferritin ≥649.95 ng/mL	<0.001^+^	0.147	0.058	<0.001^+^	<0.001^+^	0.003^+^
CEA	<0.001^+^	0.209	0.082	<0.001^+^	<0.001^+^	0.001^+^
CA125	0.765	0.902	0.339	0.046^+^	0.045^+^	0.325
CA153	0.061	0.485	0.255	0.001^+^	0.086	0.012^+^
CA724	0.016^+^	0.737	0.134	0.004^+^	0.072	0.231
NSE	<0.001^+^	0.290	0.002^+^	<0.001^+^	0.065	0.150
CYFRA21	<0.001^+^	0.082	<0.001^+^	0.002^+^	0.104	0.385
CA50	0.054	0.516	0.027^+^	0.782	0.221	0.935
CA242	0.024^+^	0.289	0.292	0.017^+^	0.236	0.305

^-^, the former is lower than the latter; ^+^, the former is higher than the latter.

ILD, interstitial lung disease; RP-ILD, rapidly progressive interstitial lung disease; LYM, lymphocyte; NLR, NEUT-to-LYM ratio; PLR, PLT-to-LYM ratio; LDH, lactate dehydrogenase; CRP, C-reactive protein; ESR, erythrocyte sedimentation rate; CADM, clinically amyopathic dermatomyositis; CDM, classic dermatomyositis.

### 3.6 Clinical characteristics of dead patients

**Figure 5:**
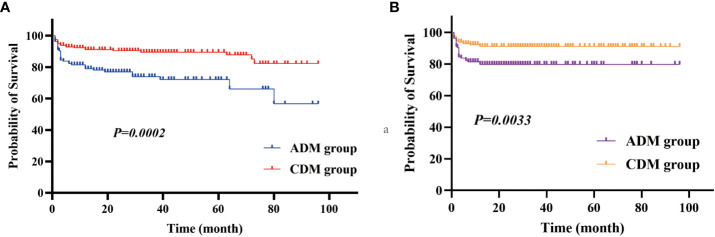
**(A)** Survival rate and **(B)** 1-year survival rate in the CADM and CDM groups.

For all patients, the median follow-up duration was 28.5 months (range: 1–100 months). The median survival period among patients with CADM was significantly shorter than that among patients with CDM (20.00 vs. 35.00 months, respectively, *P*<0.001). Overall, patients with CADM had a significantly lower survival rate (*P*<0.001, **Figure 5A**) and 1-year survival rate (*P*=0.003, **Figure 5B**) than those with CDM. A total of 57 (17.3%) patients were found to have died owing to exacerbations of ILD or infection at follow-up, whereas 44 (13.3%) died within 1 year. In order to explore the clinical differences between patients who died in the CADM and CDM groups, we further compared the clinical data of the 57 patients who died in **Table 5**. Out of the 57 deceased patients (average age: 57.81 years), 54 (94.7%) had ILD, 23 (40.4%) had RP-ILD, and 31 (54.4%) had anti-MDA5 antibodies. The rates of anti-MDA5 antibodies (*P*=0.030), ILD (*P*=0.025), RP-ILD (*P*=0.032), cough or dyspnea (*P*=0.009), and the levels of CRP (*P*=0.004) and neutrophils (*P*=0.023) in dead patients with CADM were significantly higher than those in dead patients with CDM. Conversely, the rates of Holster sign (*P*=0.023) and dysphagia in dead patients with CDM were significantly higher than those in dead patients with CADM. Notably, all patients who died in the CADM group had cough or dyspnea, but none had dysphagia. In terms of serological indices, serum ferritin and LDH levels were significantly increased, and lymphocyte levels were significantly decreased; nevertheless, no significant differences between dead CADM patients and dead CDM patients were observed. Eighteen dead patients had serum ferritin levels >2000 ng/mL; of them, 12 belonged to the CADM group, whereas 13 were accompanied by anti-MDA5 antibodies.

**Table 5:** Data of the 57 deceased patients.

	Dead patients (*N*=57)	Dead patients with CADM (*N*=35)	Dead patients with CDM (*N*=22)	*P*-value
Age at diagnosis, mean ± SD, years	57.81 ± 12.80	57.26 ± 12.40	58.68 ± 13.67	0.686
Male/female	26/31 (1:1.19)	16/19 (1:1.19)	10/12 (1:1.20)	0.985
Died within 1 year, *n* (%)	44 (77.2)	27 (77.1)	17 (77.3)	0.991
Anti-MDA5 antibodies, *n* (%)	31 (54.4)	23 (65.7)	8 (36.4)	0.030
ILD, *n* (%)	54 (94.7)	35 (100.0)	19 (86.4)	0.025
RP-ILD, *n* (%)	23 (40.4)	18 (51.4)	5 (22.7)	0.032
Cough or dyspnea, *n* (%)	53 (93.0)	35 (100.0)	18 (81.8)	0.009
Holster sign, *n* (%)	8 (14.0)	2 (5.7)	6 (27.3)	0.023
Dysphagia, *n* (%)	7 (12.3)	0 (0.0)	7 (31.8)	0.001
LYM, median (IQR), ×10^9^/L	0.65 (0.45, 0.77)	0.54 (0.45, 0.71)	0.71 (0.45, 1.06)	0.268
LDH, median (IQR), U/L	528.00 (391.50, 707.00)	481.00 (384.00, 699.70)	544.00 (409.35, 818.75)	0.372
Serum ferritin, median (IQR), ng/mL	1154.00 (700.18, 2257.95)	1281.00 (705.45, 2500.00)	1104.50 (530.75, 1756.17)	0.342
CRP, median (IQR), mg/L	18.60 (7.17, 43.00)	30.40 (9.11, 68.80)	8.26 (3.78, 20.48)	0.004
NEUT, median (IQR), ×10^9^/L	7.24 (5.31, 10.72)	8.31 (6.87, 10.79)	5.66 (3.83, 9.30)	0.023

ILD, interstitial lung disease; RP-ILD, rapidly progressive interstitial lung disease; LYM, lymphocyte; LDH, lactate dehydrogenase; CRP, C-reactive protein; NEUT, neutrophil; SD, standard deviation; IQR, interquartile range; CADM, clinically amyopathic dermatomyositis; CDM, classic dermatomyositis.

The mortality rate and 1-year mortality rate of the 29 patients with RP-ILD were 79.3% and 72.4%, respectively, of whom 23 (79.3%) belonged to the CADM group. One case was negative for any specific autoantibody, two cases were combined with an anti-PL-7 antibody, two cases were combined with an anti-PL-12 antibody, one case was combined with an anti-EJ antibody, one case was combined with an anti-OJ antibody, two cases were combined with an anti-Mi-2 antibody, one case was combined with an anti-TIF1-γ antibody, and 19 (65.5%) cases were combined with an anti-MDA5 antibodies. A total of 17 (89.5%) RP-ILD patients with anti-MDA5 antibodies died. These results indicate that patients with DM accompanied by RP-ILD had an very high mortality rate within the first year after disease diagnosis and that patients with anti-MDA5 antibodies and/or those belonging to the CADM group require urgent attention with respect to the development of RP-ILD.

Only two out of the six surviving patients with RP-ILD had combined anti-MDA5 antibodies. Surviving patients with RP-ILD had a mean LDH level of 583.67 U/L, ferritin level of 1090.62 ng/mL, and lymphocyte level of 776.67×10^6^/L on admission. Additionally, the deceased patients had a mean LDH level of 777.83 U/L, ferritin level of 2063.03 ng/mL, and lymphocyte level of 511.87×10^6^/L. Fortunately, all surviving patients were diagnosed with DM using autoantibody tests within 1 week of presentation to our department. Although these patients later developed RP-ILD, their condition improved after treatment with glucocorticoids, immunosuppressants, IVIG, and anti-infection agents, and they have survived to date. For patients with RP-ILD, we believe that relatively optimistic LDH, ferritin, and lymphocyte levels may indicate a good prognosis and that early screening for anti-MDA5 antibodies is necessary.

## 4 Discussion

This study reveals the following: (i) CADM was associated with a higher frequency of ILD and RP-ILD and a worse prognosis than CDM. (ii) Patients with anti-MDA5 antibodies and/or CADM required urgent attention to detect the development of RP-ILD. (iii) Patients with CDM were likely to have anti-NXP2 antibodies and negative myositis-specific autoantibodies, whereas those with CADM were likely to have anti-MDA5 antibodies. (iv) The presence of RP-ILD and high LDH levels were risk factors for death in CADM, but not in CDM. Mechanic’s hand was a protective factor against death in patients with CDM, but not in patients with CADM. Positive results for anti-MDA5 antibodies and high ferritin levels were risk factors for CADM and CDM. (v) Anti-MDA5 antibody-positive patients did not differ significantly, depending on whether they had CADM or CDM. (vi) When no anti-MDA5 antibody-positive patients participated, RP-ILD rates, ILD rates, and ferritin levels in patients with CADM were still higher than those in patients with CDM; whereas the mortality rates, 1-year mortality rates, and LDH levels did not differ significantly. (vii) Among patients with DM, CADM, or CDM, anti-MDA5 antibody-positive patients consistently had higher ILD and RP-ILD rates, LDH levels, lower lymphocyte levels, and worse prognoses than those who were negative for anti-MDA5 antibodies. (viii) Screening for anti-MDA5 antibodies should be the first step when suspecting the presence of CADM or CDM.

Of the 330 DM samples collected, CADM accounted for 40.9%, which was higher than that reported in previous studies (28, 29). In the comparison between CADM and CDM, we found that patients with CADM showed significantly higher anti-MDA5 antibody levels and significantly higher ILD, RP-ILD, mortality, and 1-year mortality rates than those with CDM, which were similar to the findings of a previous study (30). Additionally, we found that RP-ILD patients had anti-MDA5 antibodies and a very high probability of mortality within 1 year; furthermore, most of them had CADM. Therefore, we believe that patients with anti-MDA5 antibodies and/or CADM require urgent attention to monitor the development of RP-ILD. Surviving RP-ILD patients exhibited a significantly lower severity of anti-MDA5 antibodies and serological levels than deceased RP-ILD patients. Therefore, for patients with RP-ILD, we believe that relatively optimistic LDH, ferritin, and lymphocyte levels may indicate a good prognosis, and early screening for anti-MDA5 antibodies is necessary. Similarly, Xu et al. (31) believed that in addition to anti-MDA5 antibodies, ulcerations, serum ferritin, and lymphocyte count may aid in predicting the occurrence of RP-ILD in patients with CADM.

In our study, patients with CDM had higher rates of negative myositis-specific autoantibodies and anti-NXP2 antibodies than those with CADM, and patients with CADM had higher rates of anti-MDA5 antibodies than those with CDM. Anti-MDA5 antibodies (36.3%) were the most common specific autoantibodies in CADM, followed by anti-Jo-1, anti-PL-7, anti-EJ, and anti-PL-12, which is similar to that observed in the CADM-ILD cohort reported by Wu et al. (32).

Lymphocyte subsets in DM are thought to be reflected with ILD and disease activity to some extent (33, 34). Tumor marker levels can be used to evaluate the disease severity of DM. Additionally, CEA can be used as a noninvasive diagnostic biomarker for patients with DM-RP-ILD (35). The counts of CD3+, CD3+CD4+, CD3+CD8+, and CD16+CD56+ cells in CADM were significantly lower than those in CDM, and the levels of CEA, CA724, NSE, CYFRA21, and CA242 in CADM were significantly higher than those in CDM. Because there were insufficient data regarding the measured lymphocyte subsets and tumor marker levels of the patients, we were unable to include these two items into the prognostic risk factor prediction models of CADM and CDM. Future studies are needed to explore the impact of lymphocyte subsets and tumor marker levels on the prognosis of CADM and CDM. The prevalence of tumor events in this study was 7.6%, which is at the lower end of previously reported values (24, 36–38). During the follow-up of all patients, the probability of tumor events occurring in the CADM and CDM groups was similar to that previously reported (11, 24). At the two-year time node, Bowerman et al. (24) reported that CDM had significantly more tumor events than CADM; additionally, old age and CDM were considered independent risk factors for tumor events within 2 years of onset. Of the 25 patients with tumor-associated DM in our study, the most common antibody was the anti-TIF-1 gamma antibody (52%), similar to previous studies’ findings (39, 40).

The independent risk factors for death in CADM and CDM were evaluated using backward stepwise selection with Cox regression. In the present study, RP-ILD and high LDH levels were risk factors for death in CADM, but not in CDM. Mechanics’ hands were a protective factor against death in CDM but not in CADM; however, because of the impact of the sample size on the results, future studies are needed to confirm this. Positive anti-MDA5 antibodies and high ferritin levels are risk factors for CADM and CDM. Lian et al. (41) modeled a mortality risk score for CADM-associated ILD, identifying a ferritin level of 636 ng/mL and an LDH level of 355 U/L as the optimal clinical thresholds, similar to our results. Gan et al. (42) believed that a higher anti-MDA5 antibody titer indicated an increased likelihood of RP-ILD. Xu et al. (31) concluded that anti-MDA5 antibodies, elevated CRP levels, and decreased lymphocyte counts were independent risk factors for RP-ILD. These results further support the importance of LDH, serum ferritin, RP-ILD, anti-MDA5 antibodies, and lymphocyte levels in predicting poor outcomes.

In our study, all patients received corticosteroids during treatment. More patients with CADM received corticosteroid pulse-dose therapy, IVIG, and biological agents during treatment than those with CDM, whereas more patients with CDM maintained or even improved their condition with oral corticosteroids alone than those with CADM. In terms of the response of ILD to treatment, the presence of CADM worsened it more than that of CDM. A systematic review (43) of the treatment of MDA5-antibody-positive CADM complicated with ILD concluded that initiating combined immunosuppressive therapy early in the disease course is generally beneficial, mainly in terms of reduced morbidity and mortality. A systematic review (44) of 153 CADM cases suggested that IVIG treatment led to improvement or remission in most patients. A systematic review (45) of CADM treatment reported that most patients required more than one treatment owing to refractoriness or side effects, and IVIG was the most successful treatment. A retrospective study (46) found that IVIG is effective in the treatment of refractory cutaneous DM, enables reduction or withdrawal of immunosuppressive drugs in almost 80% of patients. Oral glucocorticoids is the first-line treatment for CDM; however, there is no consensus regarding its dosing or the addition of immunosuppressants in steroid-resistant disease. For severe CADM, the first-line therapy is antimalarials; however, it usually requires the addition of second-line cytotoxic agents. In severely refractory cases, IVIG and/or systemic calcineurin inhibitors may be employed. For severely refractory patients, IVIG and/or systemic calcineurin inhibitors are often used for treatment (47). In conclusion, DM presents a major therapeutic challenge, largely owing to our incomplete understanding of its pathogenesis and the heterogeneity of the disease itself. Randomized controlled trials are needed to determine the effects of treatment in patients with CADM. Furthermore, IVIG is the most successful treatment option for DM.

We analyzed the distribution and effects of anti-MDA5 antibodies in each group. We obtained the following findings: (i) Anti-MDA5 antibodies were prominently present in CADM. (ii) Anti-MDA5 antibody-positive patients did not show significant differences based on whether they belonged to the CADM or CDM group. (iii) When no anti-MDA5 antibody-positive patients participated, the RP-ILD rates, ILD rates, and ferritin levels in CADM were still higher than those in CDM; nonetheless, the differences decreased, whereas the mortality rates, 1-year mortality rates, and LDH levels were no longer different. (iv) Anti-MDA5 antibody-positive patients consistently exhibited higher LDH and ferritin levels, lower lymphocyte levels, higher probability of RP-ILD and ILD occurrence, and worse prognosis than anti-MDA5 antibody-negative patients, irrespective of DM, CADM, or CDM. These results indicate that anti-MDA5 antibodies not only affect the symptoms and prognosis of patients with DM but also have a non-negligible impact on the differences between CADM and CDM.

As far as we know, this is the only cohort study that enrolled more than 300 patients to compare CADM with CDM and to evaluate the distribution and impact of anti-MDA5 antibodies in these two subtypes. Nonetheless, our study still has some limitations. First, it had a retrospective design and had a sample size that was not sufficiently large. Second, myositis-specific antibody titers were not measured, and the significance of disease activity and prognosis need to be determined. Third, some patients with ILD were severely ill and unable to undergo pulmonary function tests. Fourth, the majority of our patients did not undergo pulmonary function tests owing to poor compliance or physicians’ omission. Fifth, since this is a clinical retrospective study, we only compared the medication use and prognosis of patients with CADM and those with CDM; accordingly, the comparison results are general and disordered. Despite these limitations, our study revealed differences in the clinical characteristics, antibody distribution, prognosis, and risk factors for mortality between CADM and CDM. Furthermore, it focused on the distribution and influence of anti-MDA5 antibodies in these two subtypes, Which may make clinicians view anti-MDA5 antibodies in a new light.

## Data availability statement

The original contributions presented in the study are included in the article/**Supplementary Material**. Further inquiries can be directed to the corresponding author.

## Ethics statement

The studies involving humans were approved by the Human Research Ethics Boards of The First Affiliated Hospital of Shandong First Medical University and Shandong Provincial Hospital of Traditional Chinese Medicine. The studies were conducted in accordance with the local legislation and institutional requirements. Written informed consent for participation was not required from the participants or the participants’ legal guardians/next of kin in accordance with the national legislation and institutional requirements. The animal studies were approved by the Human Research Ethics Boards of The First Affiliated Hospital of Shandong First Medical University and Shandong Provincial Hospital of Traditional Chinese Medicine. The studies were conducted in accordance with the local legislation and institutional requirements. Written informed consent was obtained from the owners for the participation of their animals in this study.

## Author contributions

QJ and DZ contributed to the collection and analysis of data. QJ, WP, YH, and ZW approved the manuscript. All authors contributed to the article and approved the submitted version.
