# Supplementary material for: Comparison of characteristics and anti-MDA5 antibody distribution and effect between clinically amyopathic dermatomyositis and classic dermatomyositis: a retrospective case-control study
Source: Front Immunol. 2023 Nov 27;14:1237209. doi: 10.3389/fimmu.2023.1237209 (PMC10720978; doi:10.3389/fimmu.2023.1237209)
Supplement: Supplementary file 1 [file Table_1.docx]

|  | DM Cohort (N=270) | ADM group (N=112) | CDM group (N=158) | P Value |
| --- | --- | --- | --- | --- |
| CD3+, median (IQR), cell/mm3 | 738.54 (489.38, 1007.26) | 502.00 (376.48, 776.70) | 882.65 (685.18, 1041.67) | **<0.001** |
| CD3+CD4+, median (IQR), cell/mm3 | 460.71 (273.115, 735.45) | 324.48 (189.74, 456.90) | 670.45 (370.97, 833.09) | **<0.001** |
| CD3+CD8+, median (IQR), cell/mm3 | 210.73 (103.34, 351.70) | 162.69 (101.33, 338.52) | 229.60 (108.23, 417.47) | **0.048** |
| CD19+, median (IQR), cell/mm3 | 170.18 (75.65, 250.00) | 175.24 (75.29, 261.36) | 153.10 (75.65, 25) | 0.953 |
| CD4+CD25+, median (IQR), cell/mm3 | 56.53 (31.79, 117.79) | 56.530 (36.65, 90.06) | 57.38 (31.79, 122.69) | 0.452 |
| CD16+CD56+, median (IQR), cell/mm3 | 121.20 (65.04, 198.83) | 89.00 (61.54, 172.61) | 125.30 (77.62, 221.96) | **0.002** |
