# Supplementary material for: Comparison of characteristics and anti-MDA5 antibody distribution and effect between clinically amyopathic dermatomyositis and classic dermatomyositis: a retrospective case-control study
Source: Front Immunol. 2023 Nov 27;14:1237209. doi: 10.3389/fimmu.2023.1237209 (PMC10720978; doi:10.3389/fimmu.2023.1237209)
Supplement: Supplementary file 2 [file Table_2.docx]

|  | DM Cohort (N=270) | ADM group (N=112) | CDM group (N=158) | P Value |
| --- | --- | --- | --- | --- |
| CEA, median (IQR), ng/mL | 2.45 (1.33, 4.52) | 3.30 (1.87, 6.61) | 2.06 (1.24, 3.48) | **<0.001** |
| AFP, median (IQR), ng/mL | 2.77 (1.78, 3.87) | 2.71 (1.84, 3.81) | 2.82 (1.77, 4.08) | 0.819 |
| CA199, median (IQR), U/mL | 11.54 (5.35, 20.05) | 11.82 (5.75, 19.71) | 10.5 (5.34, 20.56) | 0.538 |
| CA125, median (IQR), U/mL | 14.56 (9.06, 29.85) | 15.00 (9.10, 31.94) | 14.43 (9.05, 29.00) | 0.842 |
| CA153, median (IQR), U/mL | 17.32 (11.75, 28) | 17.92 (14.12, 28.03) | 17.31 (10.72, 27.03) | 0.078 |
| CA724, median (IQR), U/mL | 2.17 (1.03, 5.36) | 2.43 (1.32, 6.80) | 1.82 (0.90, 4.18) | **0.015** |
| NSE, median (IQR), ng/mL | 15.51 (11.17, 19.19) | 17.11 (12.71, 21.58) | 13.02 (10.13, 17.71) | **<0.001** |
| CYFRA21, median (IQR), ng/mL | 3.34 (2.20, 5.68) | 4.82 (3.08, 7.64) | 3.18 (2, 3.94) | **<0.001** |
| SCC, median (IQR), ng/mL | 0.70 (0.50, 1.12) | 0.70 (0.5, 1.12) | 0.69 (0.45, 1.11) | 0.479 |
| CA50, median (IQR), IU/mL | 9.38 (5.13, 15.05) | 8.79 (5.22, 11.63) | 10.78 (4.96, 23.31) | 0.051 |
| CA242, median (IQR), IU/mL | 4.81 (2.47, 8.37) | 6.50 (2.03, 10.31) | 4.10 (2.85, 5.98) | **0.019** |
