# Supplementary material for: Comparison of characteristics and anti-MDA5 antibody distribution and effect between clinically amyopathic dermatomyositis and classic dermatomyositis: a retrospective case-control study
Source: Front Immunol. 2023 Nov 27;14:1237209. doi: 10.3389/fimmu.2023.1237209 (PMC10720978; doi:10.3389/fimmu.2023.1237209)
Supplement: Supplementary file 3 [file Table_3.docx]

|  | DM Cohort (N=74) | ADM (N=39) | CDM (N=35) | P Value |
| --- | --- | --- | --- | --- |
| FVC%, mean±SD, L (btps) | 72.59±16.18 | 70.52±14.09 | 74.90±18.15 | 0.247 |
| FEV1%, mean±SD, L (btps) | 72.72±16.05 | 70.43±14.19 | 75.29±17.76 | 0.195 |
| DLCO%, mean±SD, mL/min/mm Hg | 54.83±19.31 | 54.7067±19.88 | 54.97±18.94 | 0.953 |
